# Vogt-Koyanagi-Harada Disease and COVID

**DOI:** 10.3390/jcm12196242

**Published:** 2023-09-27

**Authors:** Priscilla Manni, Maria Carmela Saturno, Massimo Accorinti

**Affiliations:** Ocular Immunovirology Service, Sapienza University of Rome, Viale del Policlinico 155, 00161 Rome, Italy; priscilla.manni@uniroma1.it (P.M.); mariacarmela.saturno@virgilio.it (M.C.S.)

**Keywords:** Vogt-Koyanagi-Harada disease, COVID-19, vaccination, uveitis, choroiditis, exudative retinal detachment, HLA DR4 antigen

## Abstract

Vogt–Koyanagi–Harada (VKH) is a rare multisystem inflammatory disease affecting the eyes, ears, brain, skin, and hair. The Coronavirus Disease 2019 (COVID-19) is a new contagious infection that might trigger the onset of VKH disease, as previously proposed for other viruses. Moreover, after the mass vaccination against SARS-CoV-2 worldwide, cases of VKH disease associated with COVID-19 vaccination have been reported. We present an overview of VKH and a comprehensive literature revision of all the VKH cases described after COVID-19 infection and vaccination, adding our experience. No differences have been found considering epidemiology and clinical findings of the disease compared to those reported in the no-COVID era. All of the patients promptly responded to systemic and local corticosteroid therapy with a good final visual prognosis. Different possible pathogenetic mechanisms underlying the onset of VKH after COVID-19 vaccination are discussed, while the presence of the HLA DR4 antigen as a genetic predisposition for the onset of the disease after COVID-19 infection and vaccination is proposed. VKH disease is one of the most frequently reported uveitic entities after COVID-19 vaccination, but a good response to therapy should not discourage vaccination. Nevertheless, ophthalmologists should be alerted to the possibility of VKH occurrence or relapse after COVID-19 vaccination, especially in genetically predisposed subjects.

## 1. Background

Vogt–Koyanagi–Harada (VKH) is a rare multisystem inflammatory disease affecting the eyes, ears, brain, skin and hair [1]. It is an autoimmune disease and several mechanisms, including infections, such as severe acute respiratory syndrome coronavirus 2 (SARS-CoV-2) and its vaccine, have been suggested as trigger factors. Coronavirus disease 2019 (COVID-19) is a new contagious infection that has a high worldwide impact on mortality and economic morbidity. This study aimed to review all reported cases of VKH disease following COVID-19 infection and vaccination, discuss the pathogenesis and add our personal experience.

## 2. Epidemiology

VKH disease is more frequent in pigmented races, such as Asians, Middle Easterners, Hispanics, and Native Americans. It is sporadic in Africa [1], and in Europe, its frequency ranges from 0% to 3% [2]. Women are affected more frequently than men, and most patients develop the disease between the second and fifth decade of life. However, pediatric cases have also been described [3,4], as well as patients with late-onset disease [5,6].

In Italy, as in other countries, VKH disease is more frequently diagnosed in women (77.7% of cases), and the age of onset is 32.6 ± 15.4 years (range 5–69 years) [7]. The age of VKH disease onset is lower in men than in women (27.8 ± 16.3 versus 34.7 ± 15.4 years). In the same cohort of patients, 18% of subjects were under 16 years old at disease onset, and 5.5% of them were younger than 10 years old [7].

## 3. Pathogenesis

Over the last few decades, several studies have clarified that the pathogenesis of VKH disease follows the pattern of autoimmune diseases. Today, although the central role of immune system dysregulation has gained definitive validation, the molecular determinants of disease onset and progression have not been satisfactorily addressed [8]. VKH disease is a T-cell autoimmune disease directed against choroidal melanocytes that express class II major histocompatibility complex (MHC) antigens, particularly against tyrosinase peptides, a melanocyte-associated enzyme involved in melanin synthesis. A robust association of VKH disease with MHC class II genes, particularly the human leukocyte antigen HLA-DR4, has been reported in different ethnic groups: for example, North American [9], Korean, Japanese [10], Italian [2], Hispanic [11], Chinese [12] and Indian patients [13]. In several reports, a higher frequency of DR4 in VKH patients was related to the HLA-DRB1*0405 allele, and in three Japanese studies, a significant increase in HLA-DRB1*0410 was also observed [14].

The immunopathological analysis of biopsies from diseased subjects suggests that eye tissues are infiltrated by specific subsets of T-cells that are supposed to target choroidal melanocytes. These findings are consistent with the extraocular manifestations of the disease that typically follow the distribution of melanocytes and the antigens they express throughout the body [1,15,16,17].

Although some controversies still exist, it has become clear that disease progression is mainly driven by CD4+ and Th17 T cell subsets that target MHC class II loaded with antigens derived from tyrosinase, tyrosinase-like proteins (TRP1-2) and melanocyte-specific antigens (e.g., MART-1, gp100) [15,17]. However, the mechanisms underscoring the loss of tolerance against these self-proteins are far from clarified, and further studies are needed to cast light on the triggering factors of disease pathogenesis that, as for many other autoimmune diseases, is a challenging issue.

One hypothesis of a possible trigger mechanism for VKH disease proposed that CD4+ T-cells sensitised to different viral peptides might cross-react with tyrosinase peptides due to a certain degree of homology (molecular mimicry). Viral infections, including hepatitis B [18], hepatitis C [19], cytomegalovirus (CMV) [20] and other herpes viruses have all been associated with VKH disease. The genome of the Epstein–Barr virus (EBV) belonging to the herpes family was detected by polymerase chain reaction (PCR) in the vitreous of patients with VKH [21]. Moreover, Sugita et al. suggested that a CMV infection may stimulate the production of T-cells and be responsible for the onset of VKH disease [20]. However, a definitive correlation of disease onset with a viral agent has not been definitively clarified, and whether a molecular mimicry phenomenon causes reactivity toward the melanocyte antigens discussed above cannot be interpreted for several reasons. For instance, the strong association of HLA class II haplotypes and CD4+ cells would argue against the existence of a virus-induced phenomenon, as these pathogens are more often linked to class I and CD8+ cells. However, it is worth recalling that the mechanisms of antigen processing and immune system modulation show a great heterogeneity (e.g., cross-presentation phenomena and splicing of peptides). 

All these considerations may apply to another environmental trigger, such as vaccination, that may contribute to the development of this autoimmune disease.

VKH onset has been reported after hepatitis, eight human papillomavirus, influenza, yellow fever and Bacillus Calmette-Guerin vaccines [18,22,23,24]. The overall incidence of vaccine-induced uveitis is 8 to 13 in 100,000 persons/year [25], and the median duration of uveitis is 346 days (range: 31–686 days) [26].

Three mechanisms of vaccine-induced uveitis have been hypothesized:

- A direct eye infection caused by a live vaccine may result in intraocular inflammation, especially in immunosuppressed patients. An example of this mechanism is the BCG vaccination against tuberculosis (TB): an in vitro model has shown that BCG can infect the retinal pigment epithelium (RPE) cells. Moreover, the effectiveness of antibiotic therapy in these forms of uveitis supports this hypothesis.
- A molecular mimicry between vaccine particles and ocular structures results in antigen-specific cell and antibody-mediated hypersensitivity reaction.
- A vaccine’s adjuvant or additives may provoke autoimmune uveitis. Adjuvants and additives enhance the host’s innate and adaptive immune response to vaccines. The immune system is directly activated against adjuvants, causing ocular inflammation and a series of clinical manifestations known as ‘Shoenfeld syndrome’, which is characterised by extraocular symptoms, such as myalgia or arthralgia, and has been described after some vaccines such as HPV, measles, mumps and rubella (MMR), influenza, diphtheria-tetanus-pertussis and BCG [27].

More recently, isolated cases of disease relapse after COVID-19 vaccinations have been reported by some studies and by us. However, the very limited sample size and the large variability in the days-after-vaccine presentation of signs and symptoms do not allow us to draw unambiguous conclusions about the relationship between the two events and the molecular factors underscoring the disease relapse. In fact, a direct connection between uveitis and drugs, including vaccines, is challenging to demonstrate in general. In 1991, Naranjo and co-workers from the University of Toronto created an algorithm, referred to as the Naranjo Scale, where it is possible to assess whether there might be a causal relationship between an identified untoward clinical event and drugs using a simple questionnaire to assign a probability score (Table 1) [28].

## 4. Clinical Features

Typical clinical features of VKH disease include bilateral choroiditis that rapidly transforms into panuveitis associated with exudative retinal detachment, meningismus associated with headaches and the pleocytosis of cerebrospinal fluid, tinnitus, hearing loss and integumentary changes, such as alopecia, poliosis and vitiligo. The latter manifestations are rarely seen during the acute stage, as they usually appear during the chronic phase of the disease depending on the effect of the treatment.

The natural course of the disease can be divided into four clinical stages:

- **Prodromal stage**: This usually lasts for 3–5 days and is characterised by nonspecific symptoms, such as malaise, fever, nausea, headache, meningismus, dizziness and orbital pain, which are sometimes followed by neurological symptoms such as cranial nerve palsies, hemiparesis, transverse myelitis and optic neuritis. During this stage, the patient may also report photophobia and tearing, as well as hair and scalp hypersensitivity [29,30].
- **Acute uveitic stage**: This appears a few days after the prodromal phase and lasts for several weeks. During this period, the patient mainly complains of blurred vision, pain and central scotoma, and most patients present with bilateral posterior uveitis. The first sign is the thickening of the posterior choroid manifested as an elevation of the peripapillary retinochoroidal layer, hyperaemia and oedema of the optic disc [31,32] and circumscribed retinal oedema. The choroidal inflammation eventually becomes multifocal with a diffuse breakdown of the RPE causing serous localised elevation of the retina that can rapidly become confluent, leading to a diffuse serous retinal detachment (SRD) [33]. The anterior segment of the eye can be affected immediately after the aforementioned clinical signs in untreated patients. It can be characterised by acute bilateral granulomatous iridocyclitis, mutton-fat keratic precipitates, iris nodules and anterior chamber shallowing due to ciliary body oedema that may lead to acute angle-closure glaucoma.
- **Chronic (or convalescent) stage**: This lasts for months or even years and results in integumentary and uveal depigmentation. Vitiligo is usually symmetrical, mainly involving the face, eyelids and trunk [1]. A slit-lamp examination may also reveal perilimbal depigmentation, as described by Sugiura, which occurs in the first month after the onset of uveitis and is mainly seen in Japanese subjects (Sugiura’s sign) [34]. During this stage, RPE scars appear in the mid-periphery of the retina. These multiple and well-defined hypopigmented lesions, sometimes surrounded by pigment, express the clinical evolution of Dalen–Fuchs nodules [34]. The natural course of the disease, especially in dark-skinned patients, is characterised by a diffuse pigment loss with a significant colour change in the fundus, which assumes a light orange-reddish appearance called ‘sunset glow fundus.’
- **Chronic recurrent stage**: This manifests as recurrent, mainly anterior granulomatous uveitis. Nevertheless, a thorough examination of the choroid at this stage with indocyanine green angiography (ICGA) or enhanced-depth imaging (EDI) and optical coherence tomography (OCT) might also find signs of active choroiditis [35]. These episodes of granulomatous uveitis are often resistant to corticosteroid therapy and may be characterised by iris mnodules, focal pigment atrophy of the iris and ocular hypotony. This is the stage where the complications of chronic inflammation, such as glaucoma, cataracts, neovascularisation of the retina and disc, subretinal fibrosis and subretinal neovascularisation, usually develop [36].

As previously described, the initial inflammatory events occur in the choroid, mainly in the choroidal stroma; therefore, VKH disease is considered a primary stromal choroiditis. Other structures, such as the retina and optic disc, become secondary to choroidal inflammation. This finding distinguishes VKH disease from other uveitis, such as sarcoidosis, which involves both the choroid and retina at random, or birdshot retinochoroiditis, which can display dual, parallel and independent involvement of the choroid and retina [1].

## 5. Extra-Ocular Manifestations

Patients with VKH disease may show extraocular involvement in the integumentary, central nervous and auditory systems. The frequency and severity of extraocular manifestation vary based on the patient’s ethnicity, which is more common in the Asian population, and on prompt and adequate treatment. If properly treated, patients do not develop complications. Integumentary manifestations in the prodromal stage include hypersensitivity to the hair and scalp. Poliosis of the eyebrows, eyelashes, scalp hair and vitiligo are typical in the chronic or convalescent stage. Vitiligo can be found in 10–63% of patients. Sugiura’s sign (perilimbal vitiligo) is the first depigmentation to appear, usually one month after the uveitis stage, but it is sporadic in Western countries. In an Italian study, skin involvement was found in 70.8% of patients with VKH. Alopecia arose in 32.94% of subjects, poliosis in 20.8% and vitiligo in 19.44%. The skin on the back or buttocks was the anatomical area initially or mainly involved. Neurologic manifestations include sterile meningitis (neck stiffness, headache), encephalitis (convulsions, altered consciousness) and cranial neuropathies (including ocular motility disturbance), particularly in the prodromal stage. Headaches are particularly and significantly different from previous manifestations by patients, and in Italy, they affect 72.2% of patients with VKH, with equal frequency in males and females [7]. Cerebrospinal fluid pleocytosis with a predominance of lymphocytes, monocytes and normal glucose has been found in more than 80% of patients with VKH and may persist for up to eight weeks. Focal neurologic signs, such as cranial neuropathies, hemiparesis, aphasia, transverse myelitis and ganglionitis, although rare, have been described [36,37,38]. Inner ear problems may affect 75% of patients, including tinnitus, deafness and vertigo. Typical cochlear hearing loss at all frequencies improves in a few months [33]. Changes caused by inner ear involvement, such as dysacusis, hearing loss and dizziness, were observed in 70% of patients, especially during the prodromal phase. Tinnitus was present in 42% of patients. The pattern of hearing loss was typically cochlear, with high-frequency involvement and improvement in two to three months. Vestibular dysfunction was rare.

## 6. Diagnosis

There is no single diagnostic test for VKH disease, and its diagnosis is mainly based on clinical features and either systemic or ocular findings. Original criteria were proposed by Sugiura in 1976 [38] and by the American Uveitis Society (AUS) in 1978 [39] (Table 2).

However, the AUS criteria failed to consider different manifestations of VKH disease at varying stages of the disease, especially the anomalies shown by fluorescein and ICGA. More comprehensive criteria were proposed in 2001 by the International Nomenclature Committee, called the Revised Diagnostic Criteria (RDC) (Table 3) [40]. RDC classifies the disease into three categories: complete (Criteria 1–5 must be present), incomplete (Criteria 1–3 and either 4 or 5 must be present) and probable (Criteria 1–3 must be present) VKH based on the presence of extraocular findings.

The Chinese Criteria, published in 2018, divided VKH into early-stage and late-stage disease, which increased the sensibility of a VKH diagnosis but required several diagnostic tests [41]. For more uniform terminology for clinical and research purposes, in March 2021, the Standardization of Uveitis Nomenclature (SUN) Working Group proposed new classification criteria. According to SUN, early-stage features include bilateral retinal detachment or characteristic uveitis with at least two of the following neurological criteria: headache, dysacusis, meningism, pleocytosis of the cerebrospinal fluid and tinnitus. Late-stage features include sunset glow fundus or characteristic uveitis with at least one of the following cutaneous findings: vitiligo, poliosis or alopecia. It is also mandatory to exclude previous ocular trauma, vitreoretinal surgery and positive treponemal syphilis serology or evidence of sarcoidosis [42].

Modern multimodal imaging has enhanced the management of uveitic diseases and VKH, and it is essential in diagnosing the extension of the disease and appropriate treatment control. Fluorescein angiography (FAG), in the early stage of the disease, shows multifocal choroiditis with multiple areas of early pinpoint hyperfluorescence followed by late leakage and pooling at the intraretinal or subretinal fluid level. In the initial phase, ICGA demonstrates a diffuse hypercyanescence, and in the intermediate and late phases, delayed choroidal perfusion and hypocyanescent lesions represent choroidal stromal inflammatory foci that cannot be highlighted by an ophthalmoscopic examination [43,44].

Fundus autofluorescence (FAF) helps evaluate the RPE and outer retinal health. In the early stage, it shows multiple hyperautofluorescences in the same areas of hyperfluorescence in FAG, corresponding to SRD. In late-stage VKH, these areas become hypoautofluorescent because of the progression of RPE damage.

EDI and OCT have demonstrated choroidal thickening in patients with early-stage VKH, even if a serous detachment is absent.

Different studies involving VKH patients undergoing corticosteroid therapy have demonstrated that choroidal thickness can be a sensitive indicator of disease changes. Specifically, it has been observed that corticosteroid treatment significantly reduced choroidal thickness in VKH patients [45,46].

EDI and OCT have demonstrated choroidal thickening in patients with early-stage VKH, even if a serous detachment is absent. This can be used to monitor the regression or progression of inflammation to adjust treatment according to the choroidal thickness. OCT-A is non-invasive multimodal imaging that provides a detailed assessment of vasculature changes. During the active phase, OCT-A demonstrates decreased choroidal and retinal vessel density, similar to the changes detectable with FAG, and focal dropout that corresponds to the inflammation areas [44,47].

## 7. Therapy

The main principle in treating early-onset VKH disease is suppressing the initial intraocular inflammation in the acute posterior uveitic/exudative stage. In the initial phase, VKH usually responds to high-dose systemic corticosteroid therapy. Treatments given early enough, from two weeks onwards when the disease is more severe, have been shown to decrease the duration of disease activity, reduce the incidence and recurrence of extraocular manifestations, improve final visual acuity outcomes and limit progression to the chronic stage [48].

The rapid discontinuation of systemic corticosteroid therapy can cause a relapse. Several studies have shown that the minimum treatment period should be six months. In their retrospective studies, Lai and Errera demonstrated the importance of a minimum of six months of systemic or immunosuppressive drug therapy to reduce the frequency and severity of relapses [49]. Rubsamen and Gass [50] demonstrated that almost all recurrences within six months of presentation were associated with too rapid or an early decrease in steroid doses. Several studies have also demonstrated that, despite proper early treatment with corticosteroid monotherapy in the acute phase, the rate of chronic recurrent inflammation in VKH ranges between 17.5% to 79% of cases, and sunset glow fundus develops [51,52,53]. Therefore, it does not seem to prevent chronic evolution in many cases.

Immunosuppressive therapy, such as methotrexate, mycophenolate mofetil, azathioprine, cyclosporine A or alkylating agents, can be added to the treatment regimen of VKH at different stages of the disease to increase the potency of corticosteroid therapy, reduce the total dosage of corticosteroid therapy needed to control the disease and when corticosteroids are contraindicated because of underlying systemic disease (e.g., diabetes). Recently, Abu El Asrar et al. found that mycophenolate, in addition to corticosteroids as a first-line therapy, leads to a significant reduction in recurrences of uveitis and the development of late complications as well as improving visual acuity in patients with VKH in the acute stage. Other authors have suggested the need to begin immunosuppressive therapy within two to three weeks after the onset of symptoms [54,55,56]. In a review article by Papasavvas, sunset glow fundus developed in only 17.5% of patients treated with combined therapy, compared to 60% of patients treated with only corticosteroid therapy [57].

These data leave no doubt that combining steroidal and nonsteroidal immunosuppression is the management of choice for initial-onset VKH disease. Considering the different ethnicities of the population reported in the studies mentioned above, the possible role of race in response to therapy cannot be excluded.

Conversely, other authors have shown that in some cases, patients can recover remarkably well after a course of corticosteroid therapy alone. Therefore, treating all subjects with VKH might lead to overtreatment, exposing patients to side effects related to immunosuppressive therapy that are potentially avoidable [58,59,60]. Additionally, a high-dose course of intravenous corticosteroids, which was eventually repeated up to three times in case ophthalmoscopic and FAG evaluation was not wholly negative, led to an excellent visual recovery with the need to treat patients with immunosuppressive drugs in only 15% of the cases [48,60]. In cases when it was impossible to treat patients from the early onset of the disease, immunosuppressive therapy was shown to reduce the incidence of sunset glow fundus, which has been associated with a higher incidence of complications and a worse visual prognosis [61].

## 8. Prognosis

The visual prognosis of patients with VKH disease has dramatically improved thanks to the use of high-dose corticosteroids, immunosuppressive drugs and advances in the management of complications such as cataracts, glaucoma and choroidal neovascularization (CNV).

Some findings have been correlated to the final visual prognosis:

Treatment.

The late initiation or delay in therapy causes more persistent inflammation, while patients with VKH disease who are adequately treated with corticosteroids have a favourable visual prognosis [62]. Treatment that lasts less than six months is significantly associated with worse final visual acuity [63]. Finally, suboptimal-dose steroid treatment in the acute phase is more likely to result in persistent inflammation [62,64]. The extent of pigmentary changes in patients appears to be associated with the dose of corticosteroids administered during the acute phase of the disease. Indeed, a high dose of corticosteroids can preserve more melanocytes and reduce the extent of damage [65].

Patient’s characteristics.

According to some authors, a worse prognosis is associated with older ages at onset [66,67]; however, according to others, the prognosis worsens in young patients [68]. Therefore, the patient’s age remains a questionable prognostic factor. HLA-DRB1*0405/0410 is more common in patients with longer disease duration. Islam studied HLA-DR4 gene variations in 46 Japanese patients, 28 of whom had prolonged disease. Significant differences were found in the variation of the DR4 gene in the two clinical subtypes. All patients with the long form had the variant DRB1*0405 or DRB1*0410. The authors concluded that as the DR4 gene variants significantly differed between the two VKH subtypes, the clinical course of VKH could be partly determined by variations in the HLA-DR gene [69].

Clinical features.

Rapid visual acuity improvement in the first phase of the disease is associated with better final visual acuity [66]. Many authors have associated initial visual acuity with final visual acuity, suggesting that eyes with better visual acuity at presentation are more likely to have better final visual acuity [30,63,66]. Furthermore, Chee et al. found that good visual acuity one month after starting treatment is a good prognostic factor, while the presence of complications is significantly associated with worse final visual acuity [66]. Finally, the number of relapses is associated with a greater risk of complications and a worse visual prognosis. A longer duration of the disease and an increase in the number of relapses expose the eye to the damaging effects of inflammation and treatment, particularly with corticosteroids.

## 9. VKH Disease and COVID Infection

In August 2023 (Health Emergency Dashboard, 2 August), there were 768,983,095 cases of COVID-19 and 6,953,743 deaths. SARS-CoV-19 is a single-stranded RNA virus with the largest known RNA genome. It is a spherical virion with an envelope consisting of phospholipids and proteins that surround the central capsid. SARS-CoV-2 belongs to the beta-coronavirus group, which also includes MERS-CoV and SARS-CoV. The latter shares approximately 75–80% of its viral genome with SARS-CoV-2 [70]. Beta-coronaviruses have three essential proteins in the pericapsid: the spike protein (S), the membrane protein (M) and the envelope protein (E). Protein S mediates virus adhesion to the cell membrane receptor, membrane fusion and viral entry into the host cell. Protein M and protein E are part of the membrane structure of the coronavirus. Another component of the beta-coronavirus is the N protein, which is a component of the helical nucleocapsid. The spike protein of SARS-CoV demonstrates an affinity for the ACE-2 receptor, which facilitates the cellular entry of SARS-CoV-2 into the host cells and represents the critical target for vaccine development.

Cases of VKH disease after a COVID-19 infection have been described, suggesting that the SARS-CoV-2 virus is a possible trigger for VKH, as previously proposed for other viruses such as hepatitis B, hepatitis C, CMV and EBV [18,19,20,21].

We performed an extensive literature analysis on PubMed from December 2020 to July 2023. The search was carried out by two authors (PM and MA) utilizing keywords such as ‘Vogt-Koyanagi-Harada disease’, ‘VKH’, ‘SARS-CoV-2’, ‘COVID-19’ and ‘COVID-19 vaccine’.

At the time of writing, the literature review includes four young patients (with a mean age of 30 ± 5.43 years) affected by VKH disease after a COVID-19 infection: the first was reported by Eliza Anthony et al. [71]. the second by Santamaria et al. [72]. the third by Saraceno et al. [73]. and the last by Juan B. Yepez [74] (Table 4). The mean time that elapsed between infection and VKH symptoms was 19.75 ± 6.57 days, and the median was 17.5 days. All the patients were women, which is consistent with women having more robust immune responses to infections than men [75,76]. The therapy used was oral steroids in all cases, with the addition of topical steroids for two cases and intravenous steroids for two cases.

## 10. VKH Disease and COVID-19 Vaccines

The association between vaccines and VKH has been reported in the literature with many types of vaccines, such as hepatitis B, influenza, yellow fever and tuberculosis vaccines. Arjun B Sood et al. described VKH onset three days after the first dose of the single-antigen hepatitis B vaccine. The vaccine contains amorphous aluminum hydroxyphosphate sulfate as an adjuvant to create a more robust immune response, primarily stimulating the innate immune response [18]. Another case of post-vaccine VKH disease was reported by Fahmeeda Murtaza et al. [24]., who diagnosed a VKH disease in a 30-year-old male. He complained of bilateral pain, redness, photophobia, floaters, headache and tinnitus, two days after receiving the inactivated vaccine against influenza. In both cases, a proposed mechanism of VKH disease after vaccination was the administration of adjuvants in genetically susceptible individuals.

A case of VKH disease associated with vaccination was also described by Campos et al. who reported VKH following yellow fever vaccination in Brazil [22]. The 34-year-old patient complained of tinnitus and eye pain two days after the vaccine, followed by decreased vision and metamorphopsia ten days post-vaccination. This live-attenuated vaccine was described as the causative agent of some mild adverse effects, such as headache, myalgia and fever, and some serious but rare adverse effects, such as multisystem disease and a neurological disease related to the attenuated virus itself. Some presumed autoimmune manifestations, such as Guillain–Barré or acute disseminated encephalomyelitis, were also described [77].

Dogal et al. reported a case of VKH disease in a 39-year-old Caucasian male after the fourth dosage of *Mycobacterium bovis* BCG [23]. BCG is made from a weakened strain of TB bacteria and provides consistent protection against the most severe forms of TB infection. It also provides a way to contrast bladder carcinoma, triggering a cytokine-mediated inflammatory response such as the case mentioned above. A complete workup for uveitis was negative (blood cell count, VDRL, TPHA, quantiferon TB gold, HIV, ESR, CRP, blood sugar concentration, creatinine, liver enzymes, urine analysis and chest X-ray) and a diagnosis of VKH was made. FAG confirmed the presence of multiple small round hypofluorescent lesions, suggesting an uneven filling of the choriocapillaris and EDI–OCT revealed multiple SRDs. The mechanism underlying the inflammation could be explained by the molecular mimicry between sequences of BCG proteins and the amino acid sequences of retinal proteins.

It is notable that different types of uveitis have been reported after vaccination. The overall incidence of all vaccine-induced uveitis was 8 to 13 in 100,000 persons/year with a preponderance of female cases [25]. The median number of days between vaccination and the onset of uveitis was 16 days (range: 1 day to 6 years; SD = 362 days) [26].

Due to the severe implications of the COVID-19 pandemic, developing a vaccine to arrest the infection has been of primary importance since the onset of the pandemic. Vaccines can be divided according to the technology used to elicit a protective immune response. The primary vaccine technologies are specified in Table 5.

Traditional vaccine strategies based on attenuated or inactivated pathogens have been shown to be highly effective for several infectious diseases [78]. However, in some situations, the whole pathogen method cannot provide the needed protection without adverse reactions, inflammation and allergic and autoimmune [79].

For vaccines based on a viral vector, genetic material from the COVID-19 virus is placed in a modified version of a different virus (viral vector). When the viral vector gets into the cells, it delivers genetic material from COVID-19 to produce copies of the S protein and stimulate the immune system against the COVID-19 virus.

Subunit vaccines only include the parts of a virus that best stimulate the immune system. When the vaccine is injected, this stimulates the immune system to produce antibodies and T-cell immunity.

mRNA vaccines use mRNA technology, which is a new type of vaccine. It works by delivering instructions to host cells inside cells for coding a viral protein, the spike protein, which acts as an antigen presented to the host immune system, eliciting an immune response and creating neutralising antibodies. Vaccines often use adjuvants, both of battery and synthetic origin, to create a more robust immune response, mainly stimulating the innate immune response. However, almost every vaccine has peculiarities that could significantly affect the efficacy or duration of immunity or the vaccine’s safety.

Cases of VKH disease have been described related to the COVID-19 vaccine, from the beginning of the vaccination campaign against COVID-19, which began in Europe on 27 December 2020, on so-called ‘Vaccine Day’, and in Italy on 31 December 2020. The literature review found five relapses following a COVID-19 vaccination and forty-six cases of new-onset VKH disease (Table 6 and Table 7, respectively).

We would like to include two additional cases in the published report, wherein two of our patients developed newly onset Vogt-Koyanagi-Harada (VKH) disease after receiving an mRNA vaccine (Tozinarem). Furthermore, one of our patients experienced a relapse of VKH after receiving the first dose of the same vaccine (Tozinarem) [82], and subsequently, this patient suffered another VKH relapse following a COVID-19 infection seven months later.

**Figure 1:**
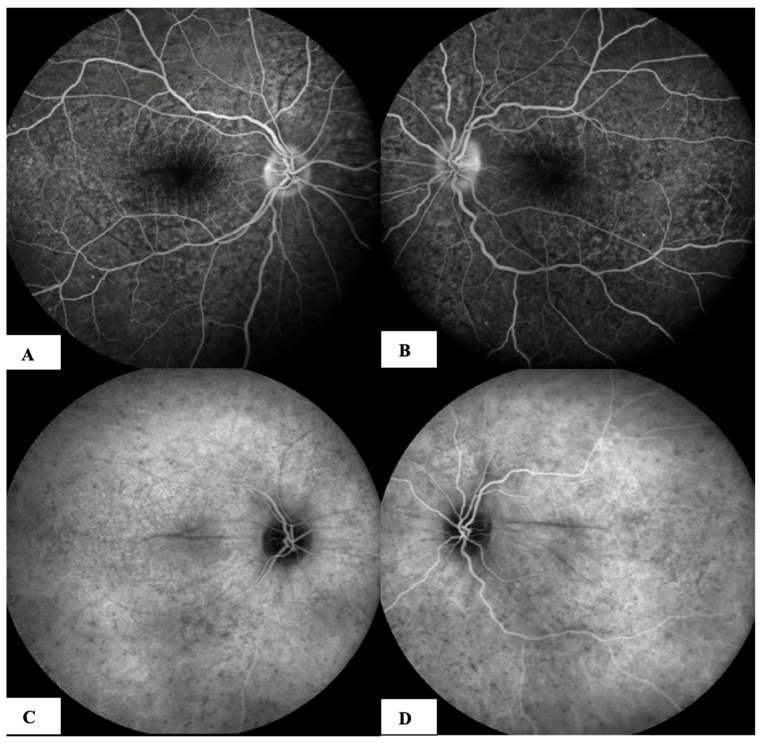
(**A**,**B**) Fluorescein angiography (FAG) after COVID vaccination: (**A**) right eye, and (**B**) left eye: typical images of pinpoint hyperfluorescence at the posterior pole and leakage of the optic nerve. (**C**,**D**) Indocyanine green angiography (ICGA) after COVID vaccination: (**C**) right eye, and (**D**) left eye, after COVID vaccination: HDDs and choroidal folds.

Patient #1 was a 49-year-old Italian woman who was referred to our clinic with a history of decreased visual acuity and metamorphopsia in both eyes, tinnitus in her left ear and an intense headache 15 days after receiving the second dose of an mRNA COVID-19 vaccine (Tozinarem). Her medical history was unremarkable, and she had no history of penetrating ocular trauma or surgery preceding the initial onset of uveitis. Her initial best corrected visual acuity (BCVA) (+0.50 sphere in the right eye and +0.25 sphere in the left eye) was 20/40, and intraocular pressure was normal. A mild reaction in the anterior chamber was found (1+ cells and flare), and diffuse foci of choroiditis with localised exudative retinal detachment and hyperaemic optic disc were detected. We performed FAG after the patient had already had cortisone boluses in another hospital and had been on oral prednisone for three days. FAG showed early choroidal perfusion inhomogeneity, dot hyperfluorescence, several areas of pinpoint hyperfluorescent foci and a mild hot disc (Figure 1A,B). ICGA showed hypofluorescent lines radiating from the optic nerve head, corresponding to choroidal folds and numerous hypofluorescent dark dots (HDDs) in both eyes appearing at an early phase and continuing well into the late angiography phase (Figure 1B,C).

OCT and EDI revealed a series of neurosensory retinal detachments and choroidal thickening. A complete work-up for uveitis, including antinuclear antibodies (ANA), anti-neutrophil cytoplasmic antibodies (ANCA), rheumatoid factor (RF), angiotensin-converting-enzyme (ACE), C-reactive protein (CPR), erythrocyte sedimentation rate (ESR), lysozyme and calcium serum levels were normal. Serology tests for the hepatitis B virus (HBV), HIV, Borrelia, Treponema pallidum and Mycobacterium tuberculosis (TBC) were negative. The nuclear magnetic resonance of the brain and cerebrospinal fluid sampling were within normal limits, while HLA typing was positive for HLA-DR4. The patient was treated with intravenous methylprednisolone (1000 mg/day) for three days followed by oral prednisone. Two weeks later, her condition improved. An ocular examination showed BCVA 20/20 in both eyes with the same correction found at disease onset, no aqueous flare and no more signs of inflammation either ophthalmoscopically detected or with macular OCT and FAG/ICG. The patient started immunosuppressive therapy with azathioprine (2 mg/kg/day) and gradually reduced corticosteroids to 5 mg, without further relapses.

**Figure 2:**
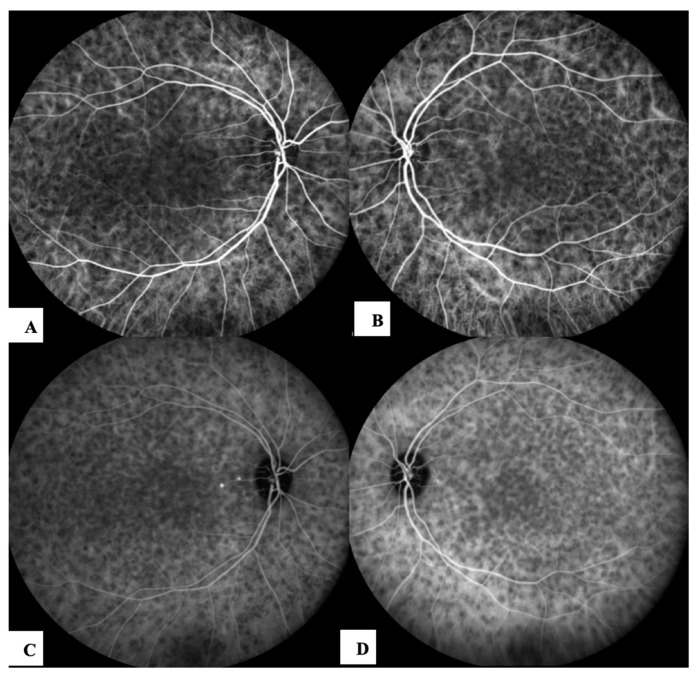
ICGA after COVID vaccination: early phase (**A**) and late phase (**C**) of the right eye, early phase (**B**) and late phase (**D**) of the left eye, showed typical hypofluorescent dark dots (HDDs).

Patient #2 was a 30-year-old Asian female referred to our clinic with a one-week history of blurred and reduced vision in both eyes, associated with severe headaches, fatigue and stiffness of the neck that occurred 14 days after receiving the first dose of an mRNA COVID-19 vaccine (Tozinarem). Her best visual acuity was bilaterally 20/20 (−0.50 sphere in both eyes), intraocular pressure was normal and an anterior segment examination revealed a mild perikeratic reaction, non-granulomatous keratic precipitates, 2+ cells and 1+ flare in the anterior chamber in both eyes. An ophthalmoscopic examination revealed 1+ vitreous haze and bilateral foci for choroiditis, disc hyperaemia and multiple retinal exudative detachments. FAG revealed multiple hyperfuorescence points at the posterior pole bilaterally in the early phase. Optic disc leakage and dye pooling in the area of the SRDs were documented in the late phase. ICGA demonstrated hypofluorescent areas of variable sizes due to choroidal active inflammation (HDDs) and fuzzy vascular patterns of large stromal vessels were more evident in the early phase of the examination (Figure 2).

OCT and EDI revealed a bilateral SRD over the swollen choroidal layer. The patient underwent a neurological examination, CT scan and MRI, which appeared normal, as did a complete work-up for uveitis. This included a complete blood count, biochemical analysis, ESR, CRP, RF, ANA and serologic screening for HIV, syphilis (Treponema pallidum hemagglutination) and TBC (purified protein derivative). The patient displayed an HLA-DR4 antigen. We decided to apply vigorous treatment to stop the inflammation, starting with intravenous methylprednisolone (1000 mg/day) for three days, followed by oral prednisolone (50 mg/day) and immunosuppressive therapy (azathioprine 2 mg/kg/day). Upon tapering the oral prednisone 20 days after starting therapy, the SR vanished, and choroidal thickness decreased. Finally, VA recovered to 20/20 in both eyes without correction. Three months after diagnosis, with prednisone (10 mg/day) and azathioprine (2 mg/kg/day), the patient was asymptomatic with a bilateral visual acuity of 20/20 in both eyes. A bilateral mild choroidal depigmentation was present, compared to the initial examination. She did not receive her second dose of the COVID-19 vaccine. Both patients were diagnosed with complete VKH disease following the diagnostic criteria for VKH disease established at the First International Workshop [40].

In both cases, there was a clear temporal relationship between the occurrence of VKH and the COVID-19 vaccination, and we calculated a score of 5 using the Naranjo adverse drug reaction probability scale (Table 1), which indicates that the symptoms observed were a possible adverse drug reaction (in this case, the vaccine) [28].

**Figure 3:**
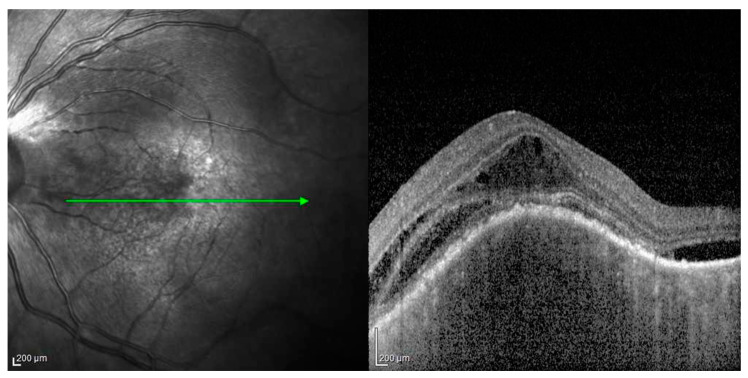
OCT after COVID infection, left eye: retinal folds and subretinal fluids and thickened choroid.

**Figure 4:**
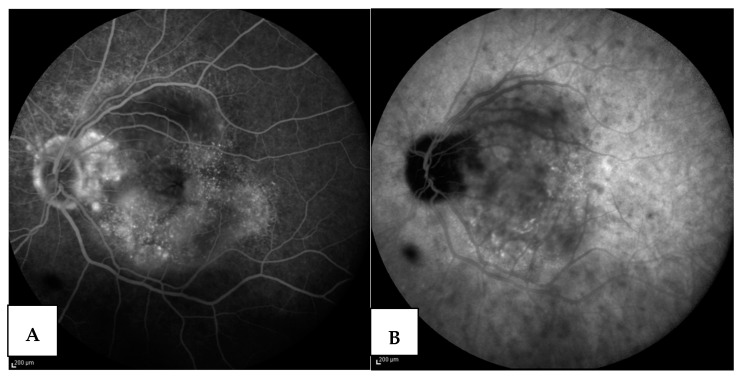
(**A**) FAG after COVID infection, left eye: typical images of pinpoint hyperfluorescence at the posterior pole with clearly defined areas of bullous retinal detachment (**B**) ICGA after COVID infection, left eye: hypofluorescent patch correspondent to area of bullous retinal detachment below, in which it is possible to observe hypofluorescent dots due to active choroidal lesions.

Patient #3 underwent a disease reactivation following both mRNA vaccines against COVID-19, already published, and as described above [82], and a COVID-19 infection. This patient was a female with VKH disease who was well controlled on azathioprine therapy (1.5 mg/kg/day) and who only presented a uveitis relapse in the right eye in May 2021, 11 days after the first dose of an mRNA vaccine for COVID-19. She received an induction of high-dose intravenous corticosteroid therapy followed by oral therapy and three orbital-floor periocular injections of triamcinolone acetonide in the right eye, which led to a complete recovery from uveitis in two weeks. Azathioprine was continued and prednisone was gradually reduced and stopped. During the six months of follow-up, no relapses occurred and she refused to take the second vaccine dose [80]. On December 2021, the patient came to us complaining of blurred and decreased vision with a central scotoma in the left eye. At that time, she was taking azathioprine (1.5 mg/kg/day). The left eye presented a VA of 20/400, 0.5 flare and cells in the anterior chamber, a hyperemic optic disc and an exudative retinal detachment with underlying choroiditis. No signs of inflammation were present in the right eye. The patient also complained of flu-like symptoms, for which a rapid test for COVID-19 turned out negative. OCT (Figure 3), fluorescein (Figure 4A) and ICGA (Figure 4B) were performed and confirmed a unilateral uveitis recurrence in the left eye.

**Figure 5:**
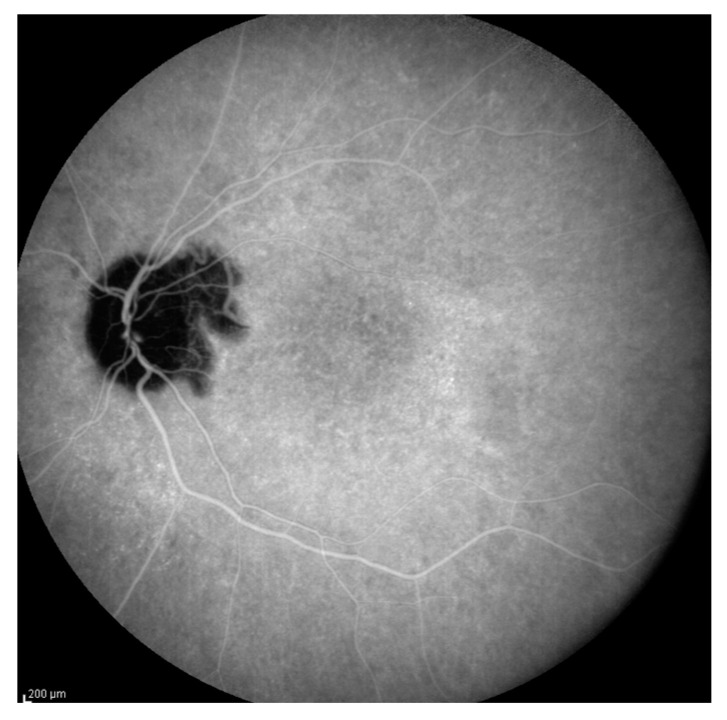
ICGA after therapy, left eye: complete resolution of all the choroidal active lesions at the posterior pole. Resolution of the overlying bullous retinal detachment.

**Figure 6:**
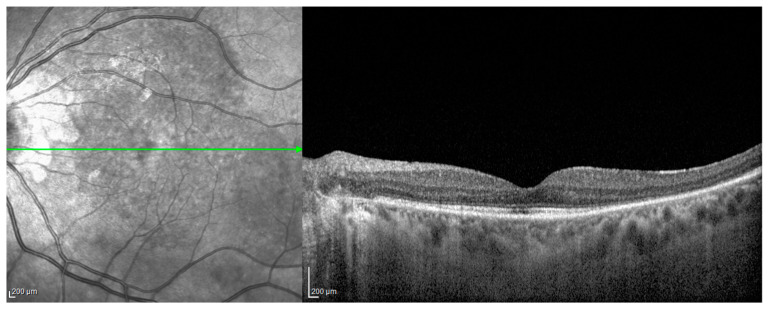
EDI OCT after therapy, left eye: reabsorption of subretinal fluid with the persistence of a slight increased choroidal thickness.

The patient was treated with oral prednisone (1 mg/kg/day), and the azathioprine dose remained the same, but because of the persistence of flu-like symptoms, a PCR test for COVID-19 was repeated and turned out positive. Therefore, azathioprine was stopped, and she was given azithromycin (500 mg/day) and oral prednisone (1 mg/kg/day) until the resolution of the flu-like symptoms. Fifteen days later, when the PCR test for COVID-19 was negative, we re-examined the patient both clinically, angiographically (Figure 5) and with EDI–OCT (Figure 6) without detecting any sign of inflammation. Visual acuity was restored to 20/20 without correction. Azathioprine was reintroduced (2 mg/kg/day) and prednisone was gradually reduced. In August 2022, while taking prednisone (7.5 mg/day) and azathioprine (2 mg/kg/day), the patient was re-infected with COVID-19 but did not complain of ocular problems. We stopped the azathioprine and increased the prednisone daily dose to 25 mg/day. Once she tested negative for COVID-19, we re-examined her and both clinical examination and angiographic studies (fluorescein and indocyanine angiography) confirmed the absence of any sign of ocular inflammation.

In summary, considering the literature review, all the relapse cases of VKH after vaccination were females, with an average age of 46.16 ± 8.78 years (range: 31–58 years), with the onset of symptoms ranging between 2 and 42 days (mean: 15.5 ± 15.62 days). They all had been vaccinated with mRNA vaccines (five with Tozinarem and one with Spikevax) and, at the time of vaccination, five out of six were on immunosuppressive therapy (one infliximab, one azathioprine, two mycophenolate and one with a combination of immunosuppressives), five without a maintenance corticosteroid therapy and one on 2.5 mg of prednisolone/day. In four cases, a complete recovery of vision was achieved after corticosteroid systemic therapy [80,81,82], in one after only topical corticosteroid therapy, while in the sixth [83], only treated with topical therapy, a recurrence of symptoms appeared after the second dose of vaccine had been administered.

Of thirty-three patients with VKH new onset after COVID-19 vaccination, thirty-one already described in the literature and two new cases reported here, the clinical findings at disease onset were those typical of the acute phase of VKH disease. These presentations included diffuse choroiditis, papillitis and the presence of subretinal fluid or bullous SRDs. The female-to-male ratio was 20:13, similar to the reported cases worldwide [18], as it was for the mean age at onset (44.12 ± 15.53 years, range: 19–78 years). Thirteen of them were vaccinated with Tozinarem (39.4%), seven (21.2%) with Vaxveria, six (18.2%) with CoronaVac, five (15.1%) with Sinopharm and two with Spikevax (6.1%). At the time of vaccination, all the new-onset cases of VKH disease were outside of any therapy.

The mean interval between COVID-19 vaccination and VKH relapses was 9.05 ± 7.67 days (range: 4 h–35 days)

All but one (96.9%) successfully responded to systemic corticosteroid therapy, one only received periocular steroids. The given therapy for two patients was not reported. Seven patients (22.6%) were treated with systemic corticosteroids (initially intravenously and then orally), six (19.3%) were directly treated with oral corticosteroids, eight (25.8%) with systemic corticosteroids associated with immunosuppressive therapy (five with azathioprine, two with methotrexate, one with cyclosporine), six (19.3%) with systemic corticosteroids and topical therapy, three (9.7%) with systemic and periocular injection and one patient (3.2%) only had one periocular injection of triamcinolone acetonide (40 mg) in both eyes.

Fifteen other patients were described in Japan, but unfortunately no specific features of these patients were reported [103].

There is no mention in the literature of the incidence of VKH disease during the COVID-19 pandemic. Between March 2020 to April 2023, in our centre, we examined ten new VKH patients, and two of them (20%) occurred after COVID-19 vaccination. Comparing these data with the pre-pandemic period, we did not find any statistically increasing incidence of VKH in our patient population.

## 11. Conclusions

VKH disease is a bilateral, diffuse granulomatous uveitis mediated by autoimmune targeting and the destruction of melanocytes. It also involves other anatomical structures that contain melanocytes, causing neurological, audiovestibular and dermatological changes [32]. Although the exact pathogenesis is still uncertain, immunological and histopathological studies suggest that an innate immune response, mediated by CD4+ T-cells against melanin and melanocytes, plays a significant role in this disease. The autoimmune response is thought to have some stimulating factors such as genetic susceptibility and viral infection [104,105].

Genetic susceptibility has been reported to be closely associated with the HLA class II antigen, HLA-DR4 [2,10,11,12,13,14]. Most probably, antigens presented within the context of MHC class II (HLA-DRB1*04:05) can activate the pro-inflammatory Th1 and Th17 cells involved in VKH disease.

VKH disease is one of the most frequently reported uveitis entities after vaccination [22,23,24], and it also appears after COVID-19 infection [71,72,73,74]. A close relationship between VKH disease and microbial infection has also been reported, with EBV, CMV, influenza A virus, mycoplasma pneumonia and TB being the supposed etiologic or inducing factors. Furthermore, with the recent SARS-CoV-2 pandemic, COVID-19 infection has also been mentioned as a trigger factor for VKH disease [71,72,73,74].

Molecular mimicry is a possible explanation for the relationship between both COVID-19 infection and vaccination. One element of a vaccine may share some antigenic features with the host, presumably a very similar epitope, such that the generation of an immune response against the foreign antigen also causes damage to host cells bearing a similar surface antigen. Talotta et al. [106]. and Akinosoglou et al. [107]. proposed that vaccination does not generate new autoimmune diseases but rather triggers long-lasting latent autoimmunity in predisposed patients, which emphasises the importance of evaluating profile risk before vaccine administration. The predisposition could be provided by HLA typing, even though the presence of HLA-DR4 was never mentioned in any of the cases published in the literature. Nevertheless, to corroborate this hypothesis, we would like to stress that all our VKH patients reported here carry the HLA-DR4 antigen: two of them presented a new onset of VKH, while the third presented two relapses, the first after a COVID-19 vaccination and the second after a COVID-19 infection.

COVID-19 infection and vaccinations are associated with a risk of various diseases. The common feature of uveitis in sarcoidosis, Behçet’s and VKH disease is that they are non-infectious inflammations with unknown pathogenesis, but with a genetic predisposition and environmental risk factors, such as bacterial and viral infections. In this context, it is possible that in all three diseases, COVID-19 infection and vaccination could lead to immune system dysregulation and the onset of ocular inflammation. Nevertheless, significantly fewer cases of Behçet’s disease and sarcoidosis have been reported after COVID-19 infection and vaccination [81,103,108,109,110,111,112,113], while it seems that Behçet patients are at higher risk of contracting COVID-19 infection, regardless of the treatment employed [114]. Therefore, a genetic background might be supposed as a co-factor in the determination of VKH onset or recurrence after COVID-19 infection or vaccination.

Considering the ongoing COVID-19 pandemic and possible new viral pandemic, if this observation is confirmed by other studies, the presence of HLA-DR4 in VKH patients should alert ophthalmologists to consider a closer follow-up of those patients who might be more prone to develop recurrences after COVID vaccination or infection.

Only eight out of thirty-one patients (25.8%) with VKH occurring after COVID vaccination were treated with immunosuppressive therapy plus corticosteroids at the onset of their disease. This finding was probably because the authors thought that immunosuppressive therapy would alter the response to the vaccination. In our two new-onset VKH patients, we used intravenous pulses of methylprednisolone (1000 mg/day) for five days, before orally administering prednisone at high doses (1 mg/kg/day). Furthermore, with one patient, we started immunosuppressive therapy (azathioprine) 15 days after the onset of the disease, while with the other patient, we started two months after.

According to the literature, using immunosuppressive drugs in the early stage of the disease helps reduce the number of relapses and improve visual prognosis, preventing the onset of sunset glow fundus [55,56]. Nevertheless, one of our patients who started immunosuppressive therapy in the acute phase of the disease in combination with a high dose of intravenous corticosteroids developed sunset glow fundus three months later. Conversely, the patient who started immunosuppressive therapy two months after the onset of the disease did not show any pigmentary changes in the fundus during a 16-month follow-up. Therefore, it is possible that immunosuppressive therapy, even when administered some months after the onset of the disease, might decrease the occurrence of sunset glow fundus, as previously reported [62].

Among patients described with a VKH reactivation after COVID-19 vaccination, all except one were on immunosuppressive therapy: two patients were treated with mycophenolate (2 mg/day), one with infliximab (5 mg/kg every 10 weeks), our case with azathioprine (1.5 mg/kg/day) and the last with a combination of immunosuppressive drugs (azathioprine, cyclosporine and mycophenolate), suggesting that immunosuppressive therapy cannot prevent a VKH relapse after vaccination.

It is interesting to note that our patient, who had disease reactivation after the vaccination and after the first COVID-19 infection, did not present a reactivation when she contracted the second COVID-19 infection. The daily dose of prednisone increased from 7.5 mg to 25 mg and immunosuppressive therapy (azathioprine 2 mg/kg/day) was immediately stopped after the COVID-19 diagnosis. A plausible hypothesis could be that corticosteroids reduce the risk of autoimmune disease reactivation, control the intense cytokine and chemokines response and improve immune dysregulation.

Finally, to better define the correlation between vaccines and VKH disease, we suggest using the Naranjo adverse drug reaction probability scale (Table 1), which has also been proposed by others [115]. In our two cases, the score was five, indicating that the observed symptoms were a possible adverse vaccine reaction.

Additionally, an important consideration is the temporal relationship between vaccination and the onset of the disease. A time range should be established within which the adverse reaction can be attributed to the drug or, as in this case, to the vaccine. From this viewpoint, a manuscript included in this review should be considered with caution because it reports quite a long time between vaccine administration and the onset of the disease. The reactivation of VKH after the second dose of the anti-SARS-CoV-2 vaccine, as described by Papasavvas, has a six-week time interval between disease manifestation and vaccination [80], while the average number of reactivation cases after non-COVID-19 and COVID-19 vaccines is 16 days and 13.5 days, respectively [26,116]. As previously mentioned, it would have a Naranjo score of 1 (only linked to the previous report associated with this reaction). Therefore, it is difficult to associate this case with vaccination.

The overall prognosis in VKH disease, whether described after vaccination or COVID-19 infection, is very good (mean visual acuity: 20/32).

Regarding the extra-ocular manifestation of VKH, no patients were described as having cutaneous involvement after a COVID-19 infection or vaccination. Vice versa, in several studies, new-onset vitiligo, one of the possible skin lesions in VKH [1], was described after COVID-19 infection and vaccination [117,118,119,120]. Vitiligo is an autoimmune disease with progressive depigmentation due to the loss of melanocytes in the epidermis. A similar pathogenesis between vitiligo and VKH might explain these data. Indeed, both diseases are thought to be related to an aberrant T-cell-mediated immune response directed against self-antigens expressed by melanocytes, the most representative of which are HMB-45, tyrosinase, S-100 protein and, therefore, the abnormal production of TNF-α and IFN-γ. We hypothesised that molecular mimicry could occur between the S protein, a component of SARS-CoV-2 used in vaccines, and HMB-45, tyrosinase and the S-100 protein [121,122]. However, in molecular analysis, the detection of peptides of at least 10 identical residues was not possible, so other components are probably involved.

In conclusion, VKH disease might occur after a COVID-19 infection and vaccination, and a relapse of the disease might also occur after both. The exact pathogenetic mechanism is not fully understood, while clinical findings are similar to those encountered and described as typical for VKH patients [1]. Patients with new onset or reactivation of VKH respond well to corticosteroid therapy and the final visual prognosis is quite good, at least in a short-term follow-up. Considering that vaccinations have helped decrease the morbidity and mortality rates of COVID-19 infection [123,124], we strongly suggest that the risks of new onset or a relapse of the disease are by far lower than those related to the unvaccinated; therefore, vaccination should be performed. On the other hand, ophthalmologists should be alerted that new-onset VKH or reactivation might occur, probably more frequently in genetically predisposed subjects. This would suggest a closer follow-up of patients with VKH who are advised to be vaccinated against COVID-19 and prompt corticosteroid therapy in those presenting a relapse. 

## Figures and Tables

**Table 1:** Naranjo Scale.

Question	Yes	No	Do Not Know	Score
1. Are there previous conclusive reports on this reaction?				
2. Did the adverse event appear after the suspected drug was administered?				
3. Did the adverse reaction improve when the drug was discontinued, or a specific antagonist was administered?				
4. Did the adverse event reappear when the drug was re-administered?				
5. Are there alternative causes (other than the drug) that could on their own have caused the reaction?				
6. Did the reaction reappear when a placebo was given?				
7. Was the drug detected in blood (or other fluids) in concentrations known to be toxic?				
8. Was the reaction more severe when the dose was increased or less severe when the dose was decreased?				
9. Did the patient have a similar reaction to the same or similar drugs in any previous exposure?				
10. Was the adverse event confirmed by any objective evidence?				

**Table 2:** American Uveitis Society Criteria.

American Uveitis Society Criteria for Diagnosis of VKH Disease
No history of trauma or surgery and one finding from at least three of the following four groups: Bilateral chronic iridocyclitis Posterior uveitis: exudative retinal detachment, formed fruste of retinal detachment (disc hyperemia or edema, subretinal macular edema) and “sunset glow” fundus Neurologic signs: tinnitus, meningismus, cranial nerve or central nervous system problem, cerebrospinal fluid pleocytosis Cutaneous sign: alopecia, poliosis or vitiligo

**Table 3:** Revised Diagnostic Criteria for VKH (2001).

Complete Vogt-Koyanagi-Harada Disease (Criteria 1 to 5 Must Be Present)
1. No history of penetrating ocular trauma or surgery preceding the initial onset of uveitis
2. No clinical or laboratory evidence suggestive of other ocular disease entities
3. Bilateral ocular involvement (A or B must be met, depending on the stage of disease when the patient is examined) A. Early manifestations of disease (1) Evidence of diffuse choroiditis (with or without anterior uveitis, vitreous inflammatory reaction or optic disc hyperemia) which may manifest as (a) Focal areas of subretinal fluid, or (b) Bullous serous retinal detachments (2) With equivocal fundus findings; both of the following must be present as well: (a) Focal areas of delay in choroidal perfusion, multifocal areas of pinpoint leakage, large placoid areas of hyperfluorescence, pooling within subretinal fluid and optic nerve staining (listed in order of sequential appearance) by fluorescein angiography, and (b) Diffuse choroidal thickening, without evidence of posterior scleritis by ultrasonography. B. Late manifestations of disease (1) History suggestive of prior presence of early findings noted in 3a and either (2) or (3) below, or multiple signs from (3). (2) Ocular depigmentation: either (a) sunset glow fundus or (b) Sugiura’s sign (3) Other ocular signs including (a) nummular chorioretinal depigmented scars, or (b) retinal pigment epithelium clumping and/or migration, or (c) recurrent or chronic anterior uveitis
4. Neurological/auditory findings (may resolve by time of evaluation) (a) Meningismus (malaise, fever, headache, nausea, abdominal pain, stiffness of the neck and back or a combination of these factors); note that headache alone is not sufficient to meet the definition of meningismus or (b) Tinnitus or (c) Cerebrospinal fluid pleocytosis.
5. Integumentary finding (not preceding onset of central nervous system or ocular disease) (a) Alopecia or (b) Poliosis, or (c) Vitiligo
Incomplete Vogt-Koyanagi-Harada disease (criteria 1 to 3 and either 4 or 5 must be present) No history of penetrating ocular trauma or surgery preceding the initial onset of uveitis, and No clinical or laboratory evidence suggestive of other ocular disease entities, and Bilateral ocular involvement. Neurologic/auditory findings; as defined for complete Vogt-Koyanagi-Harada disease above, or Integumentary findings; as defined for complete Vogt-Koyanagi-Harada disease above. Probable Vogt-Koyanagi-Harada disease (isolated ocular disease; Criteria 1 to 3 must be present) No history of penetrating ocular trauma or surgery preceding the initial onset of uveitis. No clinical or laboratory evidence suggestive of other ocular disease entities. Bilateral ocular involvement as defined for complete Vogt-Koyanagi-Harada disease above.

**Table 4:** VKH reactivation after COVID-19 infection.

	Anthon E. et al. [71].	Santamaria A. et al. [72].	Saraceno JJF. et al. [73].	Yepez J.B. et al. [74].
Age	22	32	37	29
Gender	F	F	F	F
Time interval between infection and symptoms of VKH	21 days	14 days	14 days	30 days
Treatment	Topical steroids Oral steroids	Topical steroids Oral steroids Immunosuppressive drugs	Oral steroids	Intravenous Steroids Oral Steroids

**Table 5:** COVID-19 vaccine technologies.

Live Attenuated Vaccine
Vaccine with an inactivated virus: • CoronaVac (Sinovac) • Sinopharm (China National Biotec Group) • Covaxin (Baharat Biotech
Vaccine based on a viral vector: • Johnson & Johnson (Janssen Pharmaceutical Companies) • ChAdOx1-S or Vaxzevria (Oxford and AstraZeneca)
Protein vaccines: • Nuvaxovid (Novavax) • Covovax (PTD LTD)
Messenger RNA (mRNA) vaccines: • Tozinarem or Comitnaty (Pfizer-BioNTech) • COVID-19 mRNA-1273 or Spikevax (Moderna)

**Table 6:** Cases of VKH disease reactivation after COVID vaccination.

	Papasavvas I [80].	Bolletta E [81].	Accorinti M [82].	De Domingo B [83].	Rujkorakarn P [84].
**Age**	43	44 58	55	46	31
**Gender**	F	F F	F	F	F
**Interval between vaccination and VKH symptoms onset**	6 weeks	unknown	11 days	2 days	1 week
**Type of vaccine**	Tozinarem	Tozinarem Tozinarem	Tozinarem	Tozinarem	Spikevax
**Treatment at time of vaccination**	Infliximab 5 mg/kg/10 weeks	Mycophenolate 2 g/day Mycophenolate 2 g/day	Azathioprine 1.5 mg/kg/day	none	azathioprine 2 mg/kg/day, cyclosporine 4 mg/kg/day, mycophenolate mofetil 500 mg and prednisolone 2.5 mg.
**Treatments for VKH relapses**	Oral steroids + Immunosuppressive drugs	unknown	Intravenous, oral and local steroids + Immunosuppressive drugs	Topical steroids after the first vaccination Intravenous and oral steroids after the relapse occurred following the second vaccination	Topical corticosteroids

**Table 7:** Cases of new-onset of VKH disease after COVID vaccination.

	Koong L.R [85].	Chen X [86].	Yamaguchi C [87].	Chen X [88].	Brunet de Courssou J.B [89].	Joo C.W [90].	de Queiroz Tavares Ferreira F [91].	Ding X [92].		Kim S.Y [93].	Ferreira Saraceno JJ et al. [73].
**Age**	54	19	30	57 21 28	57	50	27 39 38 32	33		72	62
**Gender**	M	F	F	F M F	F	F	M M F F	M		F	F
**Time interval between vaccination and VKH symptoms onset**	1 day	12 h	2 weeks	10 days 1 day 3 days	3 weeks	35 days	2 weeks 1 week 5 days some weeks	1 day		3 days	2 days
**Type of vaccine**	Tozinameran	CoronaVac	Tozinameran	Sinopharm CoronaVac Sinopharm	Tozinameran	Spikevax	Vaxzevria Vaxzevria Tozinameran CoronaVac	Sinopharm		Vaxzevria	Vaxzevria
**Treatment after the disease onset**	Systemic steroids (intravenous, then oral)	One periocular injection of triamcino-lone acetonide 40 mg in each eye	Systemic steroids (intravenous, then oral)	Oral steroids. Periocular steroids	Systemic steroids (intravenous, then oral) Local steroids	Oral steroids	Systemic steroids (intravenous, then oral) with immunosuppressive therapy (3 azathioprine, 1 methotrexate)	Oral steroids		Systemic steroids (intravenous and then oral)	Oral steroids
	**Reddy [94]**	**Rujkorakarn P [84]** **.**	**Sato T [95]** **.**	**Shariati MM [96]**	**Ren J. [97]**	**Han R [98]**	**Nakayama M [99]**	**Pillar S [100]**	**Wang LU [101]**	**Li Z [102]**	Yasaka Y * [103]
**Age**	30	59	45	23	46	62	53 45 29 52	24	52	48 41	78 71
**Gender**	F	M	M	F	F	M	M F M F	M	F	F M	F M
**Time interval between vaccination and VKH symptoms onset**	7 days	2 weeks	1 day	2 weeks	4 h	6 days	10 days 12 days 2 weeks 6 days	3 weeks	3 days	14 days 5 days	9 days 13 days
**Type of vaccine**	Vaxzevria	Vaxzevria	Tozinameran	Sinopharm	CoronaVac	Sinopharm	Tozinameran Tozinameran Spikevax Tozinameran	Tozinameran	Vaxzevria	CoronaVac CoronaVac	Tozinameran Tozinameran
**Treatment after the disease onset**	Oral steroids	Systemic steroids (intravenous, then oral) with immunosuppressive therapy (methotrexate)	Systemic steroids (intravenous, then oral)	Systemic steroids (intravenous) Local steroids	Systemic steroids (intravenous, then oral)	Oral steroids	Systemic steroids (intravenous) and local	Oral steroids	Systemic steroids and cyclosporine A.	Unspecified	Systemic steroids (intravenous, then oral)

* In this retrospective study, 15 additional Vogt-Koyanagi-Harada (VKH) disease cases are reported, for which the specific characteristics described in the table are not provided.

## Data Availability

Additional data of the patients cannot be provided due to privacy.
